# The physiology of foamy phagocytes in multiple sclerosis

**DOI:** 10.1186/s40478-018-0628-8

**Published:** 2018-11-19

**Authors:** Elien Grajchen, Jerome J. A. Hendriks, Jeroen F. J. Bogie

**Affiliations:** Biomedical Research Institute, Hasselt University, Diepenbeek, Belgium/School of Life Sciences, Transnationale Universiteit Limburg, Diepenbeek, Belgium

**Keywords:** Macrophage, Microglia, Polarization, Neuroinflammation, Remyelination, Multiple sclerosis

## Abstract

Multiple sclerosis (MS) is a chronic disease of the central nervous system characterized by massive infiltration of immune cells, demyelination, and axonal loss. Active MS lesions mainly consist of macrophages and microglia containing abundant intracellular myelin remnants. Initial studies showed that these foamy phagocytes primarily promote MS disease progression by internalizing myelin debris, presenting brain-derived autoantigens, and adopting an inflammatory phenotype. However, more recent studies indicate that phagocytes can also adopt a beneficial phenotype upon myelin internalization. In this review, we summarize and discuss the current knowledge on the spatiotemporal physiology of foamy phagocytes in MS lesions, and elaborate on extrinsic and intrinsic factors regulating their behavior. In addition, we discuss and link the physiology of myelin-containing phagocytes to that of foamy macrophages in other disorders such atherosclerosis.

## Introduction

Macrophages are mononuclear phagocytes that reside in every tissue of the body in which they play a crucial role in maintaining tissue homeostasis. They fulfill this task by interacting with microorganisms, remodeling tissue, and dealing with injury. Alongside their role in protective immunity and homeostasis, they also contribute to the pathology of numerous disorders. Hence, there is considerable interest in harnessing phagocyte function for therapeutic benefit, either by suppressing the activity of disease-promoting phagocytes or enhancing the mobilization of phagocyte subtypes that are advantageous. Such interventions require a thorough understanding of the spatiotemporal phenotypes that phagocytes display during disease progression.

**Fig. 1:**
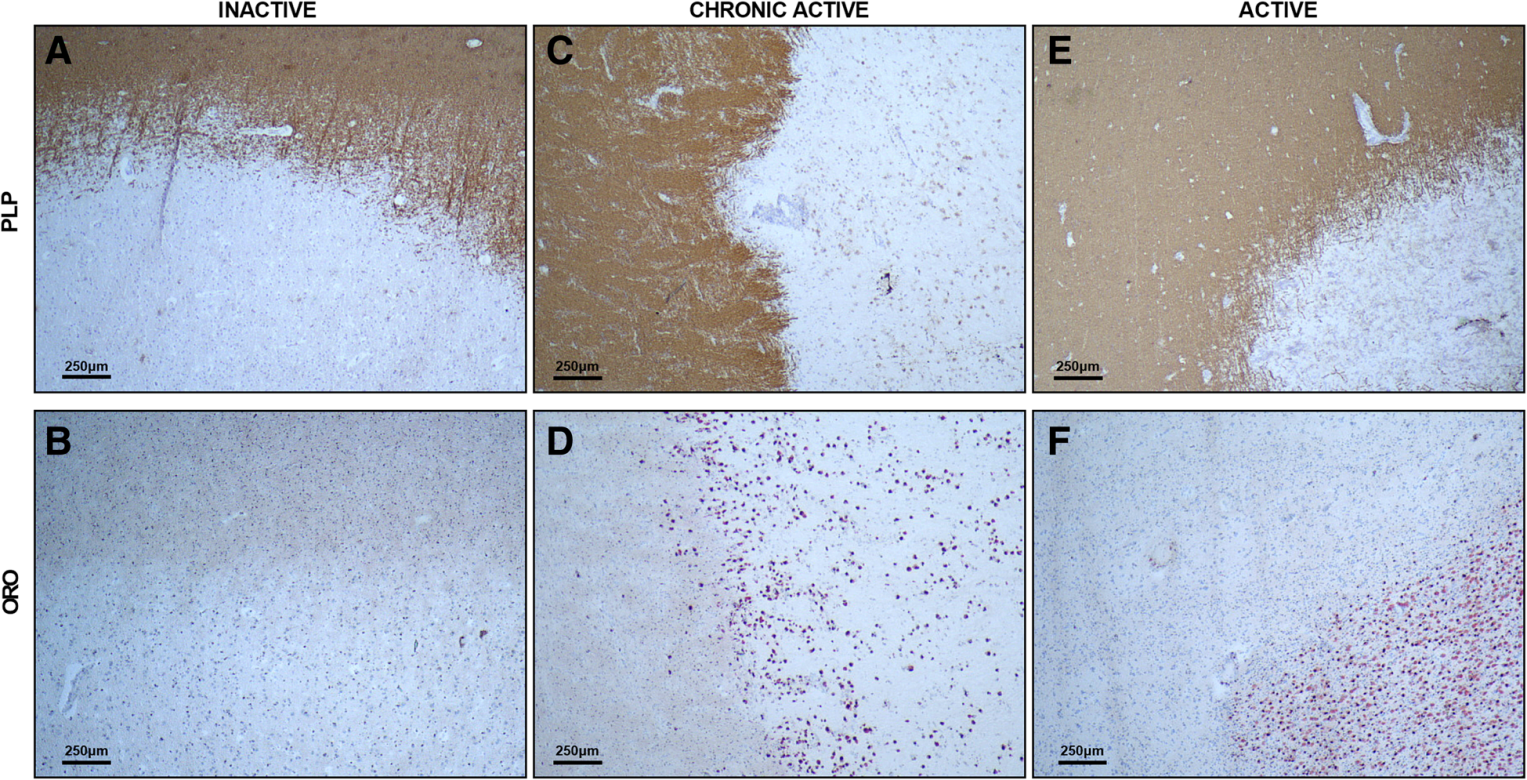
Histopathology of inactive, chronic active, and active multiple sclerosis lesions. Inactive, chronic active, and active multiple sclerosis (MS) lesions were stained for intracellular lipid droplets (oil red o; ORO) and myelin (proteolipid protein; PLP). **a** and **b**, **c** and **d**, **e** and **f** are taken from the same lesion. Foamy phagocytes (ORO^+^ cells) are apparent in demyelinating chronic active and active MS lesions, but not in inactive lesions

Multiple sclerosis (MS) is an inflammatory and neurodegenerative disease of the central nervous system (CNS) with unknown etiology. While initially regarded to be a lymphocyte-driven disorder, increasing evidence indicates that phagocytes, such as infiltrated monocyte-derived macrophages, CNS border-associated macrophages, and microglia, play an essential role in the pathogenesis of MS [14, 141]. Until recently, phagocytes were regarded to primarily cause lesion progression by releasing inflammatory and toxic mediators that negatively impact neuronal and oligodendrocyte integrity [152, 188], internalizing the intact myelin sheath [214], and presenting brain antigens to autoreactive T cells [68, 129]. However, this unambiguous concept has been challenged and it is now thought that phagocytes also have beneficial properties in MS. For example, clearance of damaged myelin is essential to facilitate CNS repair [137, 168]. Moreover, phagocytes release anti-inflammatory and neurotrophic mediators in CNS lesions and can suppress the disease-promoting activity of astrocytes and autoaggressive effector T cells [13, 18, 81, 167]. Of particular interest are myelin-containing foamy phagocytes as they make up the bulk of immune cells within active and the rim of chronic active MS lesions (Fig. 1 and [111]). Recent evidence has shed light on the many roles that these cells play in promoting and suppressing MS lesion progression, as well as the cellular mechanisms that drive their functional properties.

In this review we summarize and discuss 1) the mechanisms involved in the uptake and cellular handling of myelin, 2) the spatiotemporal phenotypes that foamy phagocytes adopt in MS patients, and 3) the intrinsic and extrinsic factors that impact the physiology of foamy phagocytes. In addition, we link the physiology of foamy phagocytes in MS to that of lipid-laden foamy macrophages in other disease such as atherosclerosis. Increasing evidence indicates that many parallels can be drawn between phagocyte subsets in various disorders.

To accomplish their functionally distinct roles in health and disease, tissue macrophages and monocyte-derived macrophages can differentiate into a spectrum of phenotypes [208]. The *ex vivo* induced M1 and M2 phenotypes represent two extremes. However, the phenotypes found *in vivo* substantially differ from these extremes. To designate the functional properties of phagocytes, we will utilize the term “M1-like” or “disease-promoting” for phagocytes that express pro-inflammatory mediators and promote MS lesion progression, and “M2-like” or “disease-resolving” for those that release anti-inflammatory and neurotrophic mediators.

## Myelin internalization

**Fig. 2:**
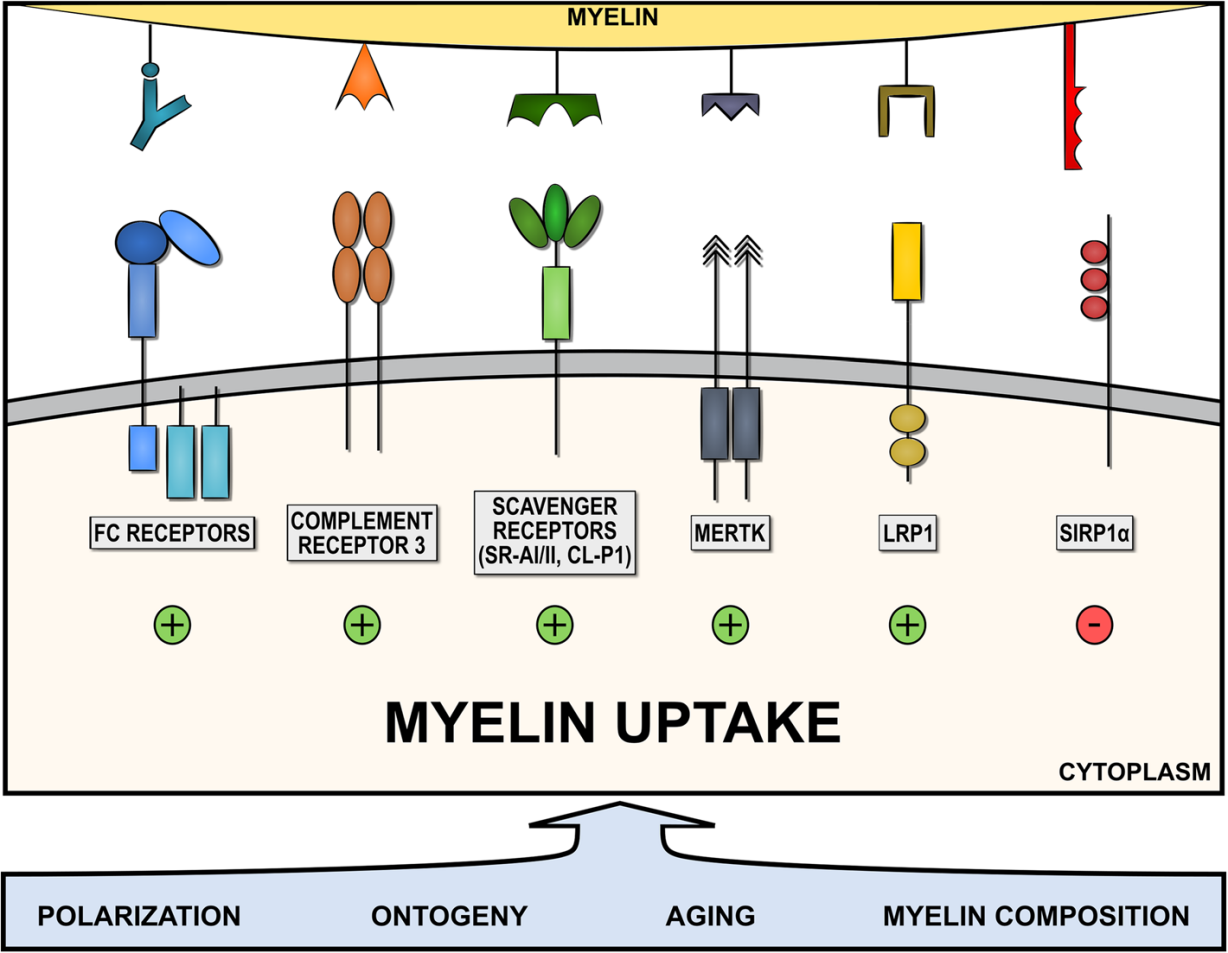
Endocytosis of myelin by phagocytes. Myelin internalization by phagocytes is dependent on the receptor repertoire as well as various intrinsic and extrinsic factors. While scavenger receptors (SR-AI/II and collectin placenta 1 (CL-P1)), Fc receptors, complement receptors (CR3), mer tyrosine kinase (MerTK*),* and low-density lipoprotein receptor-related protein 1 (LRP1) positively regulate the uptake of myelin, ligation of signal regulatory protein α (SIRPα) inhibits myelin uptake. Cell intrinsic and extrinsic factors, such as phagocyte polarization (M1- or M2-like), phagocyte ontogeny, (hematopoietic stem cells or yolk-sac progenitors), cellular aging, and myelin composition and modifications, can impact the capacity of phagocytes to internalize myelin

The uptake of myelin by phagocytes is a pathological hallmark of MS lesions and other neurodegenerative disorders. The presence of foamy phagocytes is even used as an index of MS lesion activity [160]. Initial evidence that myelin internalization largely depends on receptor-mediated endocytosis came from the observation that myelin lamellae are attached to coated pits on the macrophage surface in an animal model for MS, experimental autoimmune encephalomyelitis (EAE) [47]. Clathrin-coated pits are sites where ligand-receptor complexes cluster prior to internalization [66]. Since the discovery of receptor-mediated endocytosis of myelin, researchers have attempted to identify the culprit receptors involved in the uptake of myelin. To date, numerous receptors such as Fc, complement, and scavenger receptors are reported to drive myelin internalization. In this part of review, we elaborate on these receptors and touch upon cell extrinsic and intrinsic factors that influence myelin uptake by phagocytes (Fig. 2).

### Fc receptors

The discovery of immunoglobulin G (IgG) capping on the surface of phagocytes located amongst myelinated nerve cells in active MS lesions was the first evidence for the involvement of antibody opsonization and Fc receptors in the internalization of myelin [162]. In line with this initial discovery, a follow-up study showed that parenchymal and perivascular phagocytes in demyelinating MS lesions display a strong expression of Fc receptor I (FcRI), FcRII, and FcRIII, while microglia in the normal-appearing white matter (NAWM) barely express these receptors [192]. Subsequent *in vitro* studies confirmed the contribution of Fc receptors to the internalization of myelin by showing that opsonization of myelin with anti-myelin or galactocerebroside antibodies profoundly augments the uptake of myelin by macrophages and microglia [140, 170, 177, 179, 190]. The amount of internalized myelin was further found to depend on the degree of opsonization and the myelin epitope recognized by the antibodies [64]. However, while anti-myelin antibodies are present in the circulation of MS patients [205], serum of MS patients does not opsonize more than that of healthy controls [65]. This can be explained by the existence of anti-myelin antibodies in the sera of healthy controls, as their presence is not limited to MS patients [205]. To date, the opsonic properties of the cerebrospinal fluid (CSF) of MS patients have not been determined yet. The presence of B cell-rich meningeal follicles in the CNS of MS patients argues for the presence of a local, more concentrated, source of myelin-directed immunoglobulins in the CSF [31]. Of interest, the microenvironment also affects Fc receptor-mediated uptake of myelin. While Ig treatment was found to increase Fc receptor-mediated uptake of myelin by macrophages in a sciatic nerve model, it did not increase myelin internalization by microglia in an optic nerve model, even after addition of macrophages [112]. Follow-up studies should define if the Fc receptor expression profile on phagocytes differs in these models. In contrast to FcRI, FcRIIa, and FcRIII, FcRIIb contains an immunoreceptor tyrosine-based inhibitory motif embedded in its intracellular domain [189], which might negatively impact myelin internalization after being activated. Collectively, these studies stress the importance of Fc receptors in the uptake of myelin but also indicate that Fc receptor-mediated uptake is fundamentally different in the central and peripheral nervous system.

### Complement receptors

In addition to Fc receptors, ample evidence indicates that complement receptors are involved in the uptake of myelin by phagocytes. For instance, damaged myelin in areas of active myelin breakdown and within phagocytes colocalizes with complement components in MS lesions [2, 20, 21]. Similar, an increased density of phagocytes expressing complement receptors is observed in MS lesions [124, 204]. In particular, early studies found that the complement receptor 3 (CR3) tightly controls myelin internalization [23, 140, 164, 165, 178]. CR3 contributes to the uptake of myelin for up to 80% in the presence of active complement, while it was involved for 55-60% in the absence of active complement [164]. Counterintuitively, myelin clearance by macrophages from CR3-KO mice is not impaired [182]. A possible explanation for this discrepancy is that CR3 can both induce and reduce myelin phagocytosis at the same time. CR3 can reduce uptake of myelin by phagocytes through the activation of spleen tyrosine kinase (Syk), a non-receptor tyrosine kinase that phagocytic receptors recruit upon activation [70]. This Syk-mediated feedback mechanism was suggested to protect phagocytes from excessive intracellular accumulation of myelin. Collectively, these studies provide evidence that CR3-mediated uptake of myelin is more complex than initially regarded, being both inhibitory and stimulatory. Despite the latter studies, anti-CR3 antibodies reduce disease severity in the EAE model [85]. CR3 neutralization was found to reduce the recruitment of macrophages towards the CNS, thereby ameliorating EAE disease severity. It is tempting to speculate that a diminished phagocytic capacity may also underlie the reduced disease severity in EAE animals treated with anti-CR3 antibodies.

### Scavenger receptors

Scavenger receptors are a large family of structurally diverse proteins, which are implicated in the binding and uptake of a wide range of molecules [26, 219]. A vast amount of evidence indicates that scavenger receptors mediate the uptake of myelin. By using an organ culture model of peripheral nerves and a monoclonal blocking antibody, the scavenger receptors class AI/II (SR-AI/II) were initially found to mediate the uptake of myelin by rat macrophages [36]. At high antibody concentrations, macrophage invasion of the nerves was completely abolished, emphasizing that SR-AI/II also regulates macrophage adhesion and migration [54, 176], similar to CR3 [54, 85, 176]. Follow-up studies further defined that SR-AI/AII blocking or knockout decreases myelin uptake by mouse macrophages and microglia [49, 164, 178], and that SR-A^-/-^ mice show reduced demyelination and disease severity in the EAE model [115]. In MS lesions, SR-AI/II is highly expressed by foamy phagocytes in the rim and by ramified microglia around chronic active MS lesions [76]. This expression profile argues for the involvement of SR-AI/II in the uptake of myelin by phagocytes in MS lesions, and SR-AI/II being involved in early uptake of myelin by microglia. Aside from SR-AI/II, we recently showed that collectin placenta 1 (CL-P1), a novel class A scavenger receptor [26], also contributes to the uptake of myelin by phagocytes. In active demyelinating MS lesions, CL-P1 immunoreactivity colocalizes primarily with perivascular and parenchymal myelin-laden phagocytes. Finally, while evidence concerning its role in myelin clearance is still lacking, expression of lectin-like oxidized low-density lipoprotein receptor 1 (LOX1) is elevated at sites of active demyelination in MS lesions [76]. Future studies should define whether blockage of this class E scavenger receptor impacts myelin internalization by phagocytes.

### Other receptors

Alongside scavenger, Fc, and complement receptors, several other receptors are implicated in the endocytosis of myelin. Recently, the mer tyrosine kinase (MerTK) was found to be a functional regulator of myelin uptake by human monocyte-derived macrophages and microglia [74]. MerTK belongs to the Tyro3, Axl, and Mer (TAM) receptor family and has a hand in the internalization of apoptotic cells [114, 158]. Of interest, apoptotic cell engulfment engages a vicious cycle that leads to enhanced expression of MerTK [142, 145]. This vicious cycle depends on the intracellular activation of the lipid-sensing liver X receptor (LXR) and peroxisome proliferator-activated receptor (PPAR). Previously, we showed that myelin-containing phagocytes (mye-phagocytes) also display active LXR and PPARβ signaling [11, 15, 126]. This suggests that myelin promotes its own clearance through an LXR- and PPAR-dependent increase of MerTK. The significance of MerTK in MS pathogenesis is evidenced by the fact that polymorphisms in the MerTK gene are linked to MS susceptibility [87]. While the functional outcome of these polymorphisms remain to be clarified, they seem to depend on the genotype of individuals at HLA-DRB1 [9]*,* another MS risk gene [135]. In addition to MerTK, the low-density lipoprotein receptor-related protein 1 (LRP1) is an essential receptor for myelin phagocytosis by microglia in vitro [58]. In EAE and MS lesions, the LRP1 protein is highly expressed by phagocytes, providing evidence for involvement of LRP1 in MS pathogenesis [30, 58]. By using conditional knockout models, LRP1 deficiency in microglia but not macrophages was found to worsen EAE severity [30]. Increased EAE disease severity was associated with robust demyelination and increased infiltration of immune cells. While the authors provide evidence that microglia lacking LRP1 have a pro-inflammatory signature due to increased NF-kβ signaling, reduced microglial clearance of inhibitory myelin debris may also explain the observed effects. Collectively, these studies stress the importance of MerTK and LRP1 in the uptake of myelin by phagocytes.

### *The inhibitory* SIRPα*-CD47 axis*

Aside from receptors that stimulate myelin internalization, phagocytes also express receptors that inhibit the uptake of particles. These receptors likely evolved to limit the uptake of ‘self’ antigens or as a feedback mechanism to inhibit excessive uptake of particles. With respect to myelin internalization, signal regulatory protein α (SIRPα), a membrane glycoprotein expressed primarily by phagocytes, represents such a inhibitory receptor. Interaction of SIRPα with the “don’t eat me” protein CD47 on myelin decreases the uptake of myelin by macrophages and microglia [61, 73]. Of interest, serum also promotes an SIRPα-dependent decrease in myelin uptake irrespective of CD47 expressed on myelin [61]. A potential mechanism could be the transactivation of SIRPα by soluble SIRPα ligands present in serum. In follow-up studies, SIRPα was demonstrated to inhibit myelin internalization by remodeling of F-actin and thereby cytoskeleton function [60]. Inactivation of the paxillin-cofilin signaling axis upon SIRPα activation underlies the impact of SIRPα on cytoskeleton function and myelin uptake. Of interest, the paxillin-cofilin signaling axis also positively regulates the uptake of myelin by the scavenger, complement and Fc receptors [60, 70]. These findings place paxillin and cofilin centrally in the process of myelin internalization.

### Clearance of myelin debris

Whereas internalization of the intact myelin sheath fuels demyelination, ample evidence indicates that removal of damaged myelin debris at the lesion site promotes CNS repair. Early studies already showed that myelin contains growth inhibitory molecules such as Nogo A, which exhibit strong inhibitory effects on neurite growth and axonal regeneration [67]. Kotter et al. extended these findings by showing that myelin debris removal by phagocytes is a critical step for efficient remyelination [106]. Myelin debris was found to exert potent inhibitory effects on the ability of oligodendrocyte progenitor cells to differentiate into mature remyelinating oligodendrocytes [107, 159]. In concordance, by using the cuprizone- and lysolecithin-induced demyelination models, reduced uptake of myelin debris by macrophages and microglia resulted in inefficient axonal remyelination characterized with aberrant myelin patterns *in vivo* [113, 147, 168]. Collectively, these studies stress that clearance of myelin debris is mandatory for efficient CNS repair to progress or even initiate. Interestingly, a recent study defined that blood-derived macrophages and resident microglia have functionally divergent roles in myelin internalization. Macrophages were found to associate with nodes of Ranvier and initiate demyelination in the EAE model, whereas microglia appeared to primarily clear debris [214]. To date, the mechanisms underlying this difference remain elusive. Once identified they hold great promise for future therapeutics aimed at improving CNS repair in MS.

### Cell intrinsic and extrinsic factors influencing myelin internalization

Phagocytosis is a dynamic process involving both structural rearrangements, complex signaling events, and a plethora of phagocytic receptors. Not surprisingly, diverse intrinsic and extrinsic factors are associated with alterations in the phagocytic capacity of macrophages and microglia. For example, ample evidence indicates that the polarization status of phagocytes drives their phagocytic capacity. With respect to the latter, phagocytosis of apoptotic cells, bioparticles, and oxidized low-density lipoproteins (oxLDL) is more robust in M-CSF, IL-4/IL-10, or M-CSF/IL-10 stimulated M2-like phagocytes as compared to GM-CSF, IFNγ, or LPS stimulated M1-like phagocytes [99, 196, 222]. The uptake of myelin also matches the phenotype of macrophages and microglia. Phagocytes stimulated with the anti-inflammatory cytokines TGFβ, IL-4/IL13, IFNβ, or IL-4/IL-13/IL-10 display a higher phagocytic capacity than naïve or LPS/IFNγ stimulated M1-like phagocytes [44, 74]. These studies indicate that cytokines in the microenvironment of MS lesions, and in particular the presence of those cytokines that drive phagocyte polarization such as TGFβ, IFNγ, IL-10, and IL-4, regulate the phagocytic features of phagocytes.

A number of studies further indicate that peripheral macrophages and CNS-derived microglia differ in their capacity to internalize myelin [44, 74, 112, 140, 178]. Microglia generally show a higher capacity to internalize myelin as compared to peripheral macrophage subsets [44, 74, 140]. Differences in macrophage and microglia ontogeny, being derived from hematopoietic stem cells or yolk-sac progenitors respectively, might well explain discrepancies in their receptor expression profile and phagocytic capacity [101, 173]. On that note, both the basal and inducible expression of MerTK and myelin phagocytosis are higher in microglia as compared to monocyte-derived macrophages [74]. Likewise, we recently showed that myelin uptake increases the cell surface expression of the phagocytic receptor CL-P1 by mouse and human macrophages, but not by primary mouse microglia *in vitro* [12]*.* Finally, in contrast to peripheral macrophages, immunoglobulin treatment increases Fc receptor density on microglia [112]. Collectively, these studies suggest that differences in the density of phagocytic receptors and/or activity of signaling pathways involved in driving the expression of these receptors underlie discrepancies in the phagocytic properties of macrophages and microglia. It is also noteworthy to mention that blood-derived macrophages associate with nodes of Ranvier and initiate demyelination, whereas microglia mainly clear myelin debris [214]. This study suggests that differences in myelin uptake might also rely on the presence of receptors that recognize cryptic myelin epitopes that are not exposed on intact myelin. As phagocytosis experiments are generally carried out using myelin debris, differences in the recognition of cryptic myelin epitopes by macrophages and microglia remain to be determined.

Another factor that impacts the physiology of phagocytes is aging. Several studies indicate that aged macrophages less efficiently internalize apoptotic cells [102, 212], bacteria [75], latex beads, and opsonized sheep erythrocytes [183]. By using toxin-induced focal demyelination in the mouse spinal cord, together with heterochronic parabiosis, Ruckh et al. demonstrated that aged blood-derived macrophages also clear myelin debris less efficiently as compared to young macrophages [168]. *In vitro* experiments using mouse macrophages and microglia and human monocyte-derived macrophages confirmed that aging impairs myelin debris clearance by these phagocytes [147]. The authors further show that reduced activity of the retinoid X receptor (RXR) signaling pathway partially accounts for the observed difference in myelin uptake between young and old phagocytes. Via which pathways RXR signaling decreases the uptake of myelin by aged phagocytes remains to be clarified. While loss of RXR can directly impact the expression of phagocytic receptors such as MerTK, impaired phagocytosis can also be a mere consequence of an inability to adopt an M2-like phenotype [100]. In a follow-up study, it was demonstrated that MS-derived monocytes show a reduced uptake of myelin irrespective of the patients’ age [148]. This finding suggests that the disease state influences the phagocytic features of phagocytes in MS. It is tempting to speculate that premature innate immunosenescence, possibly due to chronic inflammation (“inflammaging”), impacts phagocyte physiology in MS patients. Increasing evidence indicates that premature aging of the immune system is apparent in MS patients [16]. Interestingly, in contrast to macrophages, aged human microglia do not show a reduction in myelin uptake compared to their younger counterparts [77]. This finding suggests that aging impact macrophages and microglia differently, and endorses the previously discussed phagocytic divergence between peripheral macrophages and CNS-derived microglia.

In addition to the polarization status, ontogeny, and aging, changes in myelin itself are reported to impact its uptake by phagocytes. Myelin isolated from MS patients is more efficiently internalized by THP-1 cells, a human monocytic cell line, and primary human microglia as compared to myelin isolated from healthy donors [77]. Enhanced uptake of myelin was not due to differences in the oxidation status of myelin. Further studies are warranted to define which modifications or changes in composition underlie the increased uptake of MS-derived myelin.

Collectively, these studies stress the complexity of myelin uptake by phagocytes, being dependent on the receptor repertoire as well as various intrinsic and extrinsic factors. Even more, while one should keep in mind that uptake of myelin debris is advantageous for CNS repair, uptake of intact myelin causes demyelination. Hence, *in vitro* studies using myelin debris should always be interpreted with caution before extrapolating to the *in vivo* situation.

## Phenotype of myelin-containing phagocytes

**Fig. 3:**
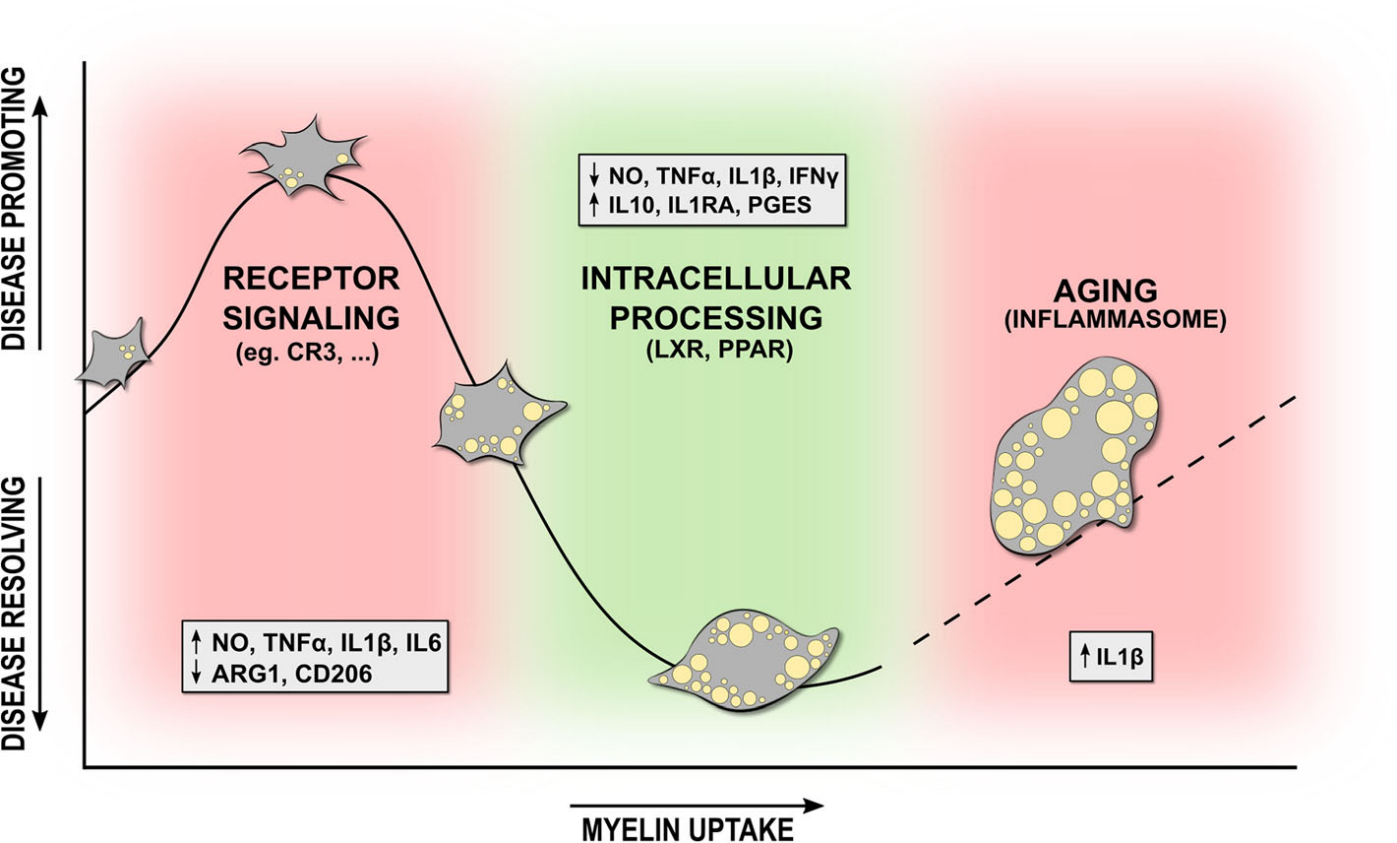
Foamy phagocyte polarization follows a triphasic pattern. Uptake of myelin initially promotes the induction of a disease-promoting phenotype of phagocytes, characterized by an increased release of inflammatory and toxic mediators, and reduced production of anti-inflammatory factors (phase I). The induction of this phenotype likely relies on the rapid activation of the FAK/PI3K/Akt/NF-κB signaling pathway following ligation of the complement receptor 3 (CR3). In time, intracellular processing of myelin will generate lipid metabolites capable of activating the anti-inflammatory liver X receptor (LXR) and peroxisome proliferator-activated receptor (PPAR). Activation of these nuclear receptors will repress the inflammatory transcriptional profile in macrophages (phase II). With aging, an inability of phagocytes to process and efflux the enormous amounts of intracellular cholesterol-rich myelin debris results in the formation of cholesterol crystals that activate the NLRP3 inflammasome (phase III)

Ample evidence indicates that myelin uptake changes the functional properties of macrophages and microglia. Some studies reported an M2-like phenotype of phagocytes upon internalization of myelin, whereas others described no effect at all, or even an M1-like activation status. In this section, we elaborate on the phenotypes of mye-phagocytes as well as the signaling pathways directing these phenotypes (Fig. 3).

The abundant presence of foamy phagocytes in MS lesions sparked interest at the end of the 20^th^ century into defining the phenotypes of these cells. In line with the prevailing dogma at that time that phagocytes merely promote lesion progression, uptake of myelin was initially demonstrated to promote the release of substantial amounts of TNFα and nitric oxide (NO) by macrophages [194]. In agreement, myelin engulfment by adult human-derived microglia induced the oxidative burst and the release of IL-1, TNFα, and IL-6 [210]. Furthermore, exposure of M-CSF stimulated M2-like macrophages to myelin debris led to a significant decrease in the expression of M2 markers and increase in the expression of markers characteristic for M1-like macrophages [203]. These studies indicate that naïve as well as pre-differentiated M2-like phagocytes adopt an inflammatory phenotype after uptake of myelin *in vitro*. Also in *in vivo* models and MS lesions, several studies defined the presence of M1-like mye-phagocytes. In the spinal cord injury (SCI) model, the accumulation of M1-like phagocytes closely correlates with the intracellular presence of myelin-derived lipids [110, 203]. Kroner and colleagues extended these findings by showing that TNFα and iron are important determinants in inducing this inflammatory phenotype of mye-phagocytes as they prevent the conversion of M1- to M2-like cells [110]. Also within MS lesions, numerous studies have demonstrated the presence of disease-promoting phagocytes in actively demyelinating lesions [14]. Interestingly, in yet another study, myelin was found to modulate microglia differentiation with a biphasic temporal pattern. Especially during the first 6h after myelin uptake, microglia display an inflammatory M1-like phenotype. However, prolonged uptake of myelin (6-24h) quenches this initial inflammatory profile of mye-microglia [121]. The speed by which myelin induces the inflammatory phenotype suggests that it ensues after rapid activation of receptor-mediated signaling pathways, instead of relying on uptake and intracellular processing of myelin. In support of this hypothesis, the myelin-induced release of inflammatory cytokines by macrophages depends on CR3 and subsequent activation of the FAK/PI3K/Akt/NF-κB signaling pathway [182]. As scavenger and Fc receptors are also closely associated with inflammatory signaling cascades [117, 219], their involvement in skewing mye-phagocytes towards a more inflammatory phenotype merits further investigation. In summary, these studies stress that, at least for a certain period of time, mye-phagocytes display an M1-like phenotype.

While early studies predominantly defined inflammatory features of mye-phagocytes, more recent studies indicate that mye-phagocytes can also acquire anti-inflammatory and wound-healing properties. Mye-phagocytes in the center of MS lesions and in *in vitro* cultures express a series of anti-inflammatory molecules while lacking pro-inflammatory cytokines [18, 220], suggesting that myelin uptake polarizes phagocytes towards an M2-like phenotype. In agreement, exposure of macrophages to sciatic or optic nerves leads to the formation of mye-macrophages that display an unique M2-like phenotype [195]. Moreover, we and others demonstrated that mye-phagocytes show a less-inflammatory phenotype in response to prototypical inflammatory stimuli, suppress autoreactive T cell proliferation, and inhibit Th1 cell polarization [11, 13, 15, 110, 121, 198]. By using adult dorsal root ganglia neurons, conditioned medium of mye-macrophages even enhanced neuron survival and neurite regeneration [81], suggesting that myelin uptake also increases the neurotrophic features of phagocytes. While studying the phenotype of mye-phagocytes, care should be taken to prevent endotoxin contamination in myelin isolates. In one study, endotoxin contamination was found to induce insensitivity to LPS in foamy macrophages [63]. Collectively, these studies indicate that myelin uptake can direct phagocytes towards an M2-like phenotype. This phenotype is shared by foamy phagocytes in other disorders, as discussed in the next sections.

Based on the assumption that myelin modulates phagocyte differentiation with a biphasic temporal pattern [121], the delayed anti-inflammatory phenotype switch of mye-phagocytes likely relies on intracellular processing of myelin-derived constituents. In line with this finding, we found that activation of the nuclear receptor LXR after myelin uptake and processing directs the less-inflammatory phenotype that mye-phagocytes display [15]. LXRs are well-known to repress an inflammatory transcriptional profile in macrophages. Moreover, LXRs are endogenously activated by cholesterol metabolites, which are abundantly present in myelin or can be formed after engulfment and processing of myelin-derived cholesterol [126]. Of interest, the deactivated phenotype of cholesterol-loaded macrophages in atherosclerotic lesions also depends in part on the LXR signaling pathway [180]*.* In addition to LXRs, we also showed that myelin-derived phosphatidylserine activates the fatty acid-sensing PPARβ/δ, thereby reducing the release of inflammatory mediators such as NO [11]. Similar to LXRs, PPARs can repress inflammatory responses mediated by NF-kβ in phagocytes. Active LXR and PPAR signaling in lesional phagocytes further emphasizes the key role that these nuclear receptors play in directing the phenotype of foamy phagocytes in MS lesions [11, 126]. Yet another study demonstrated that the p47–PHOX-mediated production of ROS after prolonged uptake of myelin represses the production of inflammatory mediators by microglia [121]. This study indicates that ROS drives a negative-feedback-circuit aimed at limiting microglia inflammation. In summary, these studies strongly suggest that the delayed anti-inflammatory phenotype of mye-phagocytes depends on signaling pathways activated after myelin uptake and processing.

Similar to the uptake of myelin, extrinsic and intrinsic factors can influence the phenotypes that mye-phagocytes adopt. For instance, a recent study demonstrated that aging skews mye-phagocytes towards an inflammatory phenotype [27]. By using the EAE and cuprizone- and lysolecithin-induced demyelination models, inflammatory foam cells harbouring large amounts of lysosomal free cholesterol were observed in old mice. An inability of aged phagocytes to process and efflux the high amounts of intracellular cholesterol-rich myelin debris appeared to underlie the accumulation of lysosomal cholesterol. In time, the accumulation of free cholesterol resulted in the formation of cholesterol crystals, which induced lysosomal rupture and activated the NLRP3 inflammasome. This study suggests that the phenotypes that foamy phagocytes display in aged individuals might even be triphasic. In addition to aging, spatiotemporal-dependent differences in the presence of cytokines are likely to impact the phenotype of mye-phagocytes differently. Future studies should define the precise cytokine milieu in active and chronic active MS lesions and determine the impact of the most abundantly expressed cytokines on the functional properties of mye-phagocytes. Finally, while both macrophages and microglia change their phenotype in a similar fashion upon myelin internalization, ontogenic differences may impact the degree of expression of the characteristic M1 and M2 markers. With respect to the latter, subtle differences have been noted in the polarization of both cell types in response to LPS, IFNγ, IL-4, and IL-13 *in vitro* [44, 59]. In depth genomic and proteomic profiling experiments may unravel differences in the phenotypes that macrophages and microglia adopt upon myelin internalization.

## Myelin-containing phagocytes in secondary lymphoid organs

While abundantly present in MS lesions, few studies demonstrated the presence of mye-phagocytes in the CNS-draining lymphoid organs of MS patients and EAE animals. De Vos et al. observed a redistribution of myelin antigens from brain lesions to cervical lymph nodes (CLNs) in primate EAE models and MS patients [38]. Antigens were found in phagocytic cells expressing MHC class II and costimulatory molecules, which were located directly juxtaposed to T cells. Likewise, by using ultrasound guided fine needle aspiration biopsy to extract cells *in vivo*, macrophages containing MBP and PLP were demonstrated in CLN of MS patients [51]. A more recent study confirmed the latter two studies and additionally showed that mye-phagocytes in CLNs of MS patients display an M2-like phenotype and express CCR7 [197]. In contrast, neuronal antigen-containing phagocytes were pro-inflammatory and did not express CCR7. These findings confirm the anti-inflammatory impact of myelin on phagocytes. Moreover, as CCR7 is crucial in lymph node-directed chemotaxis [32], this study further suggests that myelin antigens are transported to the CNS-draining secondary lymph nodes after uptake by phagocytes that subsequently migrate to CLNs by chemotaxis. However, while the increase in CCR7 on mye-phagocytes was functional *in vitro* [199], CCR7 deficiency did not alter the number of myelin-containing cells in CLNs of EAE mice compared to WT mice [197]. This implies that other chemokine receptors are involved or that myelin antigens are transported to CNS-draining lymph nodes as soluble antigens. Of interest, the recently described lymphatic vasculature in the CNS, which is connected to the deep CLNs, may lend myelin or mye-phagocytes easy access to CNS-draining lymph nodes [123]. With respect to the latter, myelin antigens and mye-phagocytes are apparent in the CSF of MS patients [105, 153], which is drained by the lymphatic vessels lining the dural sinuses [123]. Finally, after selective killing of oligodendrocytes in an *in vivo* animal model, a significant increase in intracellular lipids was found in deep CLNs, evidenced by increased Oil Red O (ORO) reactivity [122]. ORO reactivity represented intracellular myelin, as the authors also detected increased MBP and MOG levels in lumbar lymph nodes. Altogether these studies indicate that CNS demyelination coincides with the accumulation of mye-phagocytes within CNS-draining lymph nodes. How myelin antigens gain excess to these lymphoid organs, either after uptake by phagocytes that migrate by chemotaxis or as soluble particles, remains to be clarified.

To date, the pathological impact of mye-phagocytes in CNS-draining lymph nodes remains ambiguous. Mye-phagocytes in secondary lymphoid organs may present myelin antigens to autoreactive T cells, thereby driving epitope spreading and MS disease progression or even initiation [181]. Especially considering that they are located directly juxtaposed to T cells and express MHC class II and costimulatory molecules [18, 38, 50, 198]. Moreover, cervical lymphadenectomy reduces the level of brain lesions in cryolesion-enhanced EAE in rats [157]. This argues for a key role of CLNs in the induction of EAE, possibly as a site for T cell priming. In support of an immunostimulatory role of mye-phagocytes, human mye-macrophages were found to promote CD4^+^ and CD8^+^ T cell proliferation in an allogeneic mixed lymphocyte reaction and a recall response against influenza virus [198]. Macrophages treated with oxidized LDL and LDL did not impact lymphocyte proliferation, suggesting that the immunostimulatory impact is specific for myelin and not merely a hallmark of foam cells in general. Interestingly, the authors also show that mouse mye-phagocytes reduce the release of IFNγ by Th1 cells and that MOG-pulsed mye-macrophages suppress EAE severity. The latter indicates that mye-phagocytes in CLNs are not only aggressors in MS pathogenesis but can also dampen T cell-induced autoimmunity in MS. Supportive of this notion, CLNs are reported to be instrumental in the induction of intranasally induced immunological tolerance [211]. We further showed that mye-macrophages inhibit TCR-triggered lymphocyte proliferation in an antigen-independent manner *in vitro* [13]. Inhibition of T cell proliferation depended on direct contact between both cell types and the release of NO by mye-phagocytes. Interestingly, while mye-phagocytes reduced proliferation of non-myelin reactive T cells *in vivo*, they increased myelin-reactive T cell proliferation and worsened EAE severity. These findings suggest that mye-macrophages can both limit and promote T cell-induced neuroinflammation, depending on the TCR-specificity of surrounding T cells. Of note, lymph node resident CD169^+^ macrophages activate invariant natural killer T (iNKT) cells by presenting lipid antigens in a CD1d-dependent manner [3]. CD1d-restricted iNKT cells and lipid-reactive non-invariant T cells reduce neuroinflammation [39, 90]. As myelin is rich in lipids, the capacity of mye-phagocytes to activate these immune cells merits further investigation. Collectively, these studies highlight the pleiotropic impact that mye-phagocytes in CNS-draining lymph nodes may have on T cell-mediated autoimmunity in MS.

To what extent extrinsic and intrinsic factors influence the accumulation and antigen presenting capacity of mye-phagocytes in CNS lymph nodes remains to be determined. Interestingly, aging negatively impacts phagocyte migration and their antigen presenting capacity [37, 80], and therefore might well alter the ability of mye-phagocytes to home to secondary lymph nodes and present myelin-derived antigens [37]. In addition, motility seems to be differently regulated in macrophages and microglia [132], suggesting that ontogenic differences might also be involved. On that note, while both macrophages and microglia express CCR7 [42, 199], differences in the expression of other chemokine receptors such as CX3CR1 and CCR2 are reported between microglia and specific peripheral monocyte subsets [14]. Interestingly, the transmembrane chemokine CX3CL1 is induced in inflamed lymphatic endothelium and dendritic cell-specific deletion of CX3CR1 markedly delays lymphatic trafficking [94]. These findings suggest that CX3CR1^hi^ microglia are more prone to home to secondary lymph nodes in MS than CX3CR1^lo^ monocyte subsets. However, more research is warranted to certify the abovementioned claims.

## Parallels with foamy macrophages in other disorders

**Fig. 4:**
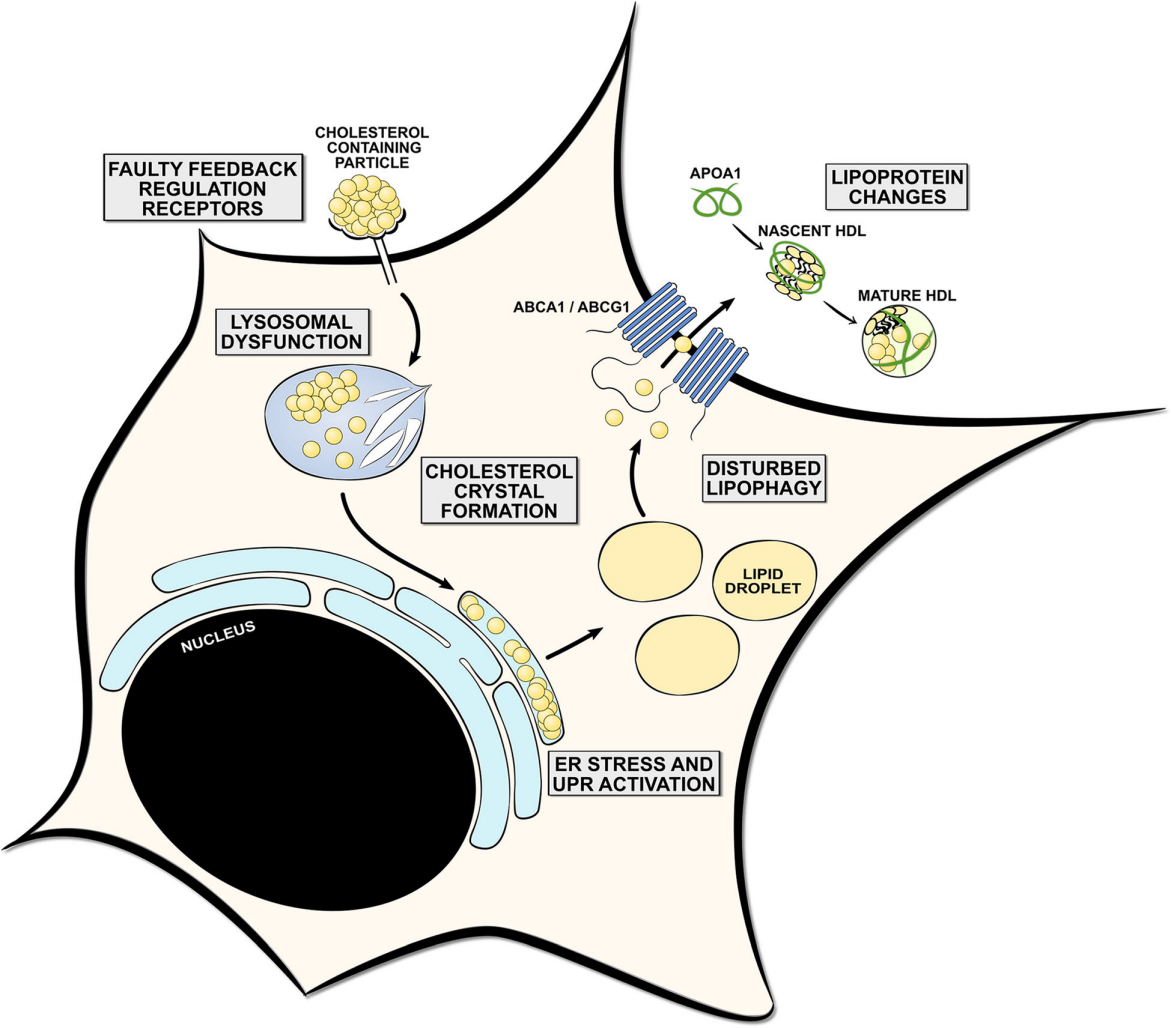
Homeostatic and dysfunctional processing of cholesterol-containing lipid particles. During homeostasis, phagocytes are well equipped to cope with relatively minor increases of cholesterol. However, sustained intracellular accumulation of cholesterol, as observed in in many peripheral pathologies and following infections with persistent pathogens, can lead to disturbances in pathways that mediate the degradation, storage, or efflux of cholesterol. First, faulty feedback regulation of phagocytic receptors may result in an uncontrolled uptake of cholesterol-containing lipid particles. Second, lysosomal cholesterol accumulation can result in lysosomal dysfunction by reducing lysosomal acidification and causing lysosomal leakiness. In addition, sustained accumulation of cholesterol can lead to the formation of cholesterol crystals that activate the caspase-1-activating NLRP3 inflammasome. Third, persistent cholesterol trafficking to ER membranes can trigger ER stress and the unfolded protein response (UPR). Fourth, dysfunctional lipophagy machinery can hamper the capacity of foamy phagocytes to process cholesterol within lipid droplets, thereby impeding the cells’ capacity to dispose of intracellular cholesterol. Finally, quantitative and qualitative changes in lipoproteins can impact the capacity of foamy phagocytes to efflux cholesterol. Altogether, disturbances in the abovementioned pathways are well-known to promote the induction of a disease-promoting phenotype of foamy phagocytes and eventually even cause apoptosis. While ample evidence suggests that faulty regulation of these pathways also occurs in myelin-containing phagocytes, more research is warranted to define to what extent they impact their inflammatory features

Myelin-containing phagocytes are a pathological hallmark of CNS disorders such as MS. However, foamy macrophages packed with lipid bodies are also abundantly present in many peripheral pathologies associated with chronic inflammation, such as atherosclerosis and non-alcoholic steatohepatitis (NASH), and following infections with persistent pathogens like *Mycobacterium tuberculosis* (Mtb), *Chlamydia pneumoniae*, and *Toxoplasma gondii* [98, 139, 161, 169, 213]. Especially in atherosclerosis, foamy macrophage physiology has been thoroughly investigated. In atherosclerotic lesions, macrophages acquire a foamy appearance through the uptake and degradation of native and modified lipoproteins, such as oxLDL. Generally, macrophages are well equipped to cope with minor intracellular increases of LDL. However, sustained intracellular accumulation of LDL-derived lipids leads to disturbances in pathways that mediate the degradation, storage, and efflux of these lipids. As a consequence, macrophages become engorged with lipids and obtain a disease-promoting phenotype. In this section, we discuss and link the malfunctioning of these pathways to the development and physiology of phagocytes that internalized the lipid-rich myelin sheath (Fig. 4).

### Uncontrolled internalization of myelin

In atherosclerosis, the swift removal of modified LDL from the intima provides protection against its cytotoxic and damaging effects. However, continuous uptake of modified LDL by macrophages also promotes the formation of inflammatory, lipid-engorged, foamy macrophages, which eventually may be an even more harmful event. Diverse studies suggest that feedback regulation of receptors involved in the uptake of modified LDL goes awry in atherosclerosis. For example, the expression of receptors involved in the uptake of modified LDL, such as CD36 and SR-A, remains high throughout lesion development in atherosclerosis [138]. Similar to atherosclerosis, uptake of myelin may also be a continuous process in neurodegenerative disorders. This is supported by the finding that the expression of receptors involved in the uptake of myelin, such as FcRIII, SR-AI/II, and MerTK, is elevated in active MS lesions [76, 206]. We further demonstrated that myelin uptake results in the activation of LXRs and PPARβ/δ [11, 15, 126]. Both nuclear receptors induce the expression of MerTK and opsonins, such as C1qa, and C1qb [142, 145]*.* This increase in expression may augment the internalization of myelin by phagocytes in demyelinating disorders. Of interest, continuous activation of PPARγ by modified oxLDL may promote a similar vicious cycle of LDL uptake in oxLDL-loaded macrophages by inducing the expression of CD36 [89, 146]. Furthermore, we demonstrated that oxidized myelin more potently increases the expression of the phagocytic scavenger receptor CL-P1 compared to unmodified myelin [12]. More importantly, while CL-P1 surface expression gradually decreases on macrophages treated with unmodified myelin, macrophages exposed to oxidized myelin retain a high expression of CL-P1 over time. These findings indicate that unmodified and oxidized myelin impact macrophage function differently, similar to native and oxLDL. In addition, they suggest faulty feedback regulation of CL-P1 when phagocytes are exposed to oxidized forms of myelin. While counter regulatory processes that inhibit myelin internalization such as the CD47/SIRPα axis exist [61], CD47 was found to be decreased at the mRNA level and expressed at low abundance on protein level in MS lesions [73]. Even more, microRNA profiling of MS lesions identified modulators of the regulatory protein CD47 [96, 187]. Reduced signaling through this inhibitory CD47/SIRPαinhibitory pathway may further boost myelin uptake and demyelination. Collectively, these studies stress that faulty regulation of phagocytic and inhibitory receptors in MS lesions can lead to the uncontrolled internalization of myelin by phagocytes.

### Lysosomal dysfunction

Ample evidence indicates that lysosomal dysfunction is a critical step in the formation of M1-like foam cells and disease progression in atherosclerosis and NASH [78]. The sequestration of LDL-derived free cholesterol within lysosomes is regarded to underlie lysosomal dysfunction and the induction of M1-like macrophages in these disorders [7, 33, 46, 92, 116, 191, 218]. In MS patients, an increase in several lysosomal enzymes is apparent in plaques, periplaque areas, NAWM, and CSF samples [35, 45, 72], which indicates active breakdown of lipids and other macromolecules in the CNS. Strikingly, while active MS lesions are packed with metabolically active mye-phagocytes, lysosomal function or dysfunction within these cells remains largely uninvestigated. Free cholesterol is the predominant form of cholesterol in myelin. Hence, continuous uptake of myelin by phagocytes is likely to result in lysosomal accumulation of free cholesterol and consequently lead to lysosomal and phagocyte dysfunction. Interestingly, an early study using the EAE model demonstrated that abnormalities in lysosomal permeability are apparent before the development of clinical and histological changes [56]. Similar, cerebral lysosomes seem to be more fragile in MS white matter compared to white matter of healthy controls [128]. Lysosomal abnormalities equally affected the plaque, periplaque, and NAWM in MS patients. These studies suggest that lysosomes in the CNS of MS patients are more prone to become dysfunctional. A more recent study showed that aged mye-phagocytes have a tendency to accumulate large amounts of lysosomal cholesterol [27]. Lysosomal accumulation of myelin-derived cholesterol led to the activation of NLRP3 inflammasome. Despite these studies, it remains unclear to what extent lysosomal dysfunction occurs in foamy phagocytes in MS lesions, and what the impact of lysosomal accumulation of myelin-derived cholesterol is on lysosomal integrity.

LDL loading is reported to downregulate the expression of Niemann Pick Disease type C1 and C2 (NPC1 an NPC2) in macrophages. NPC1 and NP2 are membrane proteins that facilitate the transfer of free cholesterol from lysosomes to the endoplasmic reticulum (ER) for further processing [91]. Hence, a reduced expression of NPC1 and NPC2 can augment lysosomal free cholesterol sequestration and lysosomal dysfunction. While no studies defined changes in the expression of NPC1 and NPC2 in phagocytes upon myelin uptake, fingolimod (FTY720), which is currently used for treatment of MS, increases the expression of NPC1 and NPC2 on both mRNA and protein level in NPC mutant fibroblasts [150]. Likewise, FTY720 increases the expression of NPC1 in human macrophages and improves their survival after sustained lipid uptake [10]. This increase in NPC expression may boost the trafficking of free cholesterol to the ER in mye-phagocytes, thereby counteracting the accumulation of free cholesterol in lysosomes and preventing lysosomal dysfunction. Thus, apart from blocking the egress of leukocytes from secondary lymph nodes [22], FTY720 can suppress MS lesion progression by restoring or retaining lysosomal function in mye-phagocytes.

Whereas it is generally assumed that lysosomal dysfunction is a secondary event in the pathophysiology of atherosclerosis and NASH, a genome-wide association study identified polymorphisms in the gene encoding the lysosomal enzyme galactocerebrosidase (GALC) in MS patients [87]. This argues for lysosomal dysfunction being a potential primary pathological event in MS. In Krabbe disease, lack of GALC activity results in lysosomal accumulation of galactosylcerebrosides and galactosphingosine in phagocytes and oligodendrocytes, leading to severe demyelination [103]. Haematopoietic stem cell transplantation corrects the metabolic defect in Krabbe disease, which indicates the importance of dysfunctional GALC in leukocytes in disease pathogenesis [109]. To what extent polymorphisms in the GALC gene impact lysosomal function and lipid accumulation in phagocytes in MS upon myelin uptake remains to be clarified. In summary, several studies suggest that lysosomal dysfunction can occur in mye-phagocytes in MS lesions. However, more in-depth studies examining the abovementioned lysosomal parameters in *in vitro* cultured mye-phagocytes and within MS lesions are warranted to certitude this claim.

### Formation of cholesterol crystals and inflammasome activation

Sustained accumulation of cholesterol within foamy macrophages in atherosclerosis, NASH, and following Mtb infections results in the formation of cholesterol crystals [6, 25, 88]. Several studies indicate that cholesterol crystals destabilize lysosomes, thereby activating the caspase-1-activating NLRP3 inflammasome and promoting the release of IL-1β [43, 55, 82, 119]. Similar to foamy phagocytes in these disorders, phagocytes accumulate copious amounts of cholesterol *in vitro* and *in vivo* following uptake of myelin [12, 126]. Moreover, several studies demonstrated the presence of cholesterol crystal-like structures in mye-phagocytes [8, 27, 113]. By using electron microscopy imaging, numerous mononuclear cells containing degenerated myelin were found to accumulate needle-shaped cholesterol structures in late stages of Wallerian degeneration [8]. Cholesterol crystals are also apparent in IBA1^+^ mye-microglia in the corpus callosum of cuprizone-treated animals [113]. Finally, a more recent study showed that aging results in the accumulation of cholesterol crystals in mye-phagocytes, leading to NLRP3 inflammasome activation [27]. To date, it remains unclear whether cholesterol crystals are also formed in foamy phagocytes within MS lesions, and to what extent inflammasome activation in these cells impacts MS lesion progression. With respect to the latter, inflammasome activation is apparent in the CNS and peripheral cells in several neurodegenerative disorders [79, 83, 136, 156]. Furthermore, mice lacking NLRP3, caspase-1, or IL-18 exhibit reduced neuroinflammation, demyelination, and neurodegeneration [69, 79, 86, 93, 125, 215, 216], which underscores the pathogenic role for the inflammasome in neurodegenerative disorders. Notably, predominantly macrophages and microglia produce IL-1β in EAE and MS lesions [24, 193], arguing for phagocytes being the culprit cells involved in the abovementioned knockout models. Of particular interest, the scavenger receptor CD36 is closely associated with the *de novo* formation of intracellular cholesterol crystals and NLRP3 inflammasome activation in oxLDL-loaded macrophages [175]. Hence, CD36 may well fulfill a similar function in mye-phagocytes [49]. More in-depth studies are needed to define if *de novo* formation of cholesterol crystals underlies inflammasome activation within mye-phagocytes or if lysosomal destabilization due to the free cholesterol accumulation causes inflammasome activation.

### ER stress and the unfolded protein response

The ER plays a key role in the biosynthesis, processing, and trafficking of proteins. Environmental factors or elevated protein synthesis can lead to the accumulation of misfolded or unfolded proteins in the ER, also called ER stress. ER stress triggers the unfolded protein response (UPR), which attempts to restore ER homeostasis by attenuating global protein synthesis and degrading unfolded proteins. If the UPR fails to restore ER homeostasis, apoptotic signaling pathways are activated to remove stressed cells [202].

ER stress and UPR activation are known to occur in oxLDL-loaded macrophages *in vitro* and macrophages in human atherosclerotic lesions and apoE-knockout mice [144, 217, 221]. Moreover, cholesterol trafficking to ER membranes in cholesterol-loaded macrophages results in UPR activation and promotes phagocyte apoptosis [41, 53]. Similar to atherosclerosis, ER stress and UPR activation is apparent in MS and EAE lesions. An increased mRNA and protein expression of activating transcription factor 4, CCAAT-enhancer-binding protein homologous protein, calreticulin, X-box-binding protein 1, and immunoglobulin-heavy-chain-binding protein was found in NAWM and demyelinating lesions of MS patients [34, 71, 130, 134, 143, 151]. Interestingly, calreticulin colocalizes with ORO^+^ phagocytes in MS lesions, which points towards ER stress and UPR activation in mye-phagocytes [151]. Likewise, foamy phagocytes in active MS lesions show an increased expression of the mitochondria-associated membrane protein Rab32, which is closely associated with the UPR [71]. Active UPR signaling is also observed in phagocytes, T cells, astrocytes, and oligodendrocytes during the course of EAE [28, 40, 131, 151]. Importantly, inhibition of the UPR using crocin reduces ER stress and the inflammatory burden in EAE animals. The reduced EAE disease severity was paralleled with preserved myelination and axonal density, and reduced immune cell infiltration and phagocyte activation [40]. This study underscores the detrimental impact of ER stress and the UPR on neuroinflammation and neurodegeneration. Remarkably, despite ER stress and UPR activation, no studies have reported the presence of apoptotic and necrotic foamy phagocytes in active demyelinating MS lesions yet. Phagocyte apoptosis might be difficult to detect histologically, owing to the fact that dying cells are rapidly cleared by neighboring phagocytes through efferocytosis [209]. Thus, while studies point towards a role for ER stress and UPR activation in MS pathology, more research is warranted to define the underlying mechanisms, culprit cell types, and functional outcome.

### Disturbed autophagy/lipophagy

Autophagy is a catabolic process essential for cellular and tissue homeostasis. While it is crucial for the degradation of dysfunctional and unwanted proteins and organelles, increasing evidence indicates that it also controls lipid degradation, a process called lipophagy [120]. Ouimet et al. defined that lipophagy plays a key role in cholesterol efflux from lipid-laden macrophages [154]. During lipophagy, autophagosomes and lysosomes fuse with lipid droplets after which esterified cholesterol is hydrolyzed by specific enzymes, such as lysosomal acid lipase, into free cholesterols. Unlike esterified cholesterol, free cholesterol is a substrate for ABCA1 and ABCG1-mediated efflux to apoA-I or HDL, respectively. Hence, active lipophagy represents a way to dispose intracellular cholesterol, thereby preventing their intracellular accumulation.

Autophagy is tightly linked to the pathogenesis of MS. However, the precise role that autophagy plays in the pathogenesis of MS and to what extent the autophagy machinery is dysfunctional is poorly understood. To date, the majority of studies have focused on the impact of autophagy on lymphocyte survival and homeostasis in MS [1, 48, 108]. However, autophagy likely also impacts foamy phagocyte function in MS lesions. As autophagy regulates the antigen presenting capacity of dendritic cells [5], future studies should define whether is it also involved in the presentation of myelin antigens by foamy phagocytes locally in the CNS and secondary lymphoid organs. Similar, the influence of autophagy/lipophagy on lipid efflux by foamy phagocytes merits further investigation, in particular with respect to aging. Recently, aging was reported to hamper the efflux efficacy of mye-phagocytes in diverse animal models for demyelination [27]. Malfunction of the lipophagy machinery may underlie the age-related discrepancy in the capacity of foamy phagocytes to dispose of intracellular cholesterol. Of interest, increasing evidence suggests that dysfunctional autophagy is apparent in foamy macrophages in atherosclerosis, and contributes to lipid accumulation, apoptosis, and inflammasome hyperactivation in these cells [118, 163]. As autophagy regulates phagocytosis by modulating the expression of phagocytic receptors [17], defining the impact of autophagy on the uptake of myelin also deserves further attention. Thus, while increasingly being acknowledged to impact MS disease progression, more research is warranted to define the role that autophagy plays in directing the functional properties of foamy phagocytes, and elucidate whether the autophagy machinery becomes dysfunctional in phagocytes engorged with myelin-derived lipids.

### Lipoprotein alterations and modifications

While we focused in the previous sections on intracellular processes going awry in cholesterol-loaded foamy phagocytes, extracellular factors such as lipoproteins can also impact lipid processing, thereby directing the physiology of these foamy macrophages [172]. Generally, high levels of LDL, and in particular modified forms of LDL, drive the inflammatory activation of macrophages after sustained uptake, thereby promoting lesion formation and progression in atherosclerosis, as described in the previous sections. In contrast, high-density lipoproteins (HDL) have anti-atherogenic properties, which are attributed to their crucial role in reverse cholesterol transport [166]*.* Ample evidence suggests that lipoprotein levels, subclasses, and function are also altered in MS patients and associated with disease activity. For instance, disability in MS patients is positively correlated to plasma LDL, apoB, and total cholesterol levels [127, 186, 207]. Furthermore, MS patients display elevated oxLDL levels in plasma and the CNS [149, 155], and an increase in plasma auto-antibodies directed against oxLDL [4]. In contrast to LDL, controversy exists regarding HDL levels in MS patients. Whereas some studies demonstrated a decrease [133], other showed no change or even an increase in HDL levels [4, 62, 171]. Of note, we recently reported that distinguishing between different HDL subclasses is of importance when investigating HDL levels [95]. Irrespective of these studies, higher serum HDL levels correlate with reduced blood-brain barrier injury and a decreased infiltration of immune cells into the CSF of MS patients [52]. We recently identified an altered lipoprotein profile in relapsing-remitting MS (RR-MS) patients, especially in low-BMI RR-MS patients, with modified and dysfunctional HDL [95]. By using LC-MS/MS, we demonstrated that HDL is modified at its ApoA-I tyrosine and tryptophan residues. Such modifications are increasingly being acknowledged to alter HDL function [104, 166, 174]. Specifically, the Trp50 and Trp72 domains are responsible for the initiation of lipid binding to ApoA-I [84, 201]. This suggests that in in low-BMI RR-MS patients, cholesterol efflux may be dysfunctional, potentially leading to the inflammatory accumulation of myelin-derived lipids in mye-phagocytes. In line with this hypothesis, serum HDL of RR-MS patients less efficiently accepts cholesterol via the ABCG1 transporter compared to serum HDL of healthy controls [95]. To what extent tyrosine and tryptophan modifications underlie changes in HDL functionality in RR-MS patients remains to be clarified. Altogether, these studies strongly suggest that quantitative and qualitative changes in lipoproteins are apparent in MS and can impact phagocyte lipid load and physiology. In particular, the function of phagocytes containing abundant myelin-derived lipids can potentially be severely compromised by these changes in LDL and HDL.

### Protective foamy macrophages

Similar to MS, increasing evidence indicates that the phenotype of lesional macrophages in atherosclerosis is more complex than previously thought [184]. For a long time, sustained cholesterol uptake and consequent disturbances in metabolic pathways were believed to promote the induction of inflammatory, pro-atherogenic macrophages, as discussed in the previous paragraphs. However, a regulated accumulation of desmosterol following cholesterol loading also suppresses the inflammatory activation of foamy phagocytes in the absence of overt inflammation [180]. Of interest, desmosterol was found to mediate its effects on the macrophage phenotype by activating LXRs. Likewise, another study demonstrated that oxLDL loading increases the expression of the typical M2 marker arginase I in a PPAR-dependent manner [57]. These findings suggest that LDL accumulation can also suppress inflammation and may limit lesion progression when the inflammatory burden in atherosclerotic lesions is low. In line with these studies, considerable phenotypic variation of macrophages is apparent in atherosclerotic lesions [19, 29, 97, 185]. Strikingly, similar to oxLDL-loaded macrophages, we found that myelin-derived lipids skew phagocytes towards a less inflammatory phenotype in LXR- and PPAR-dependent manner upon uptake of myelin [11, 15]. Also, within MS lesions considerable phenotypic variation is observed [18, 200]. Boven et al. demonstrated that foamy phagocytes in the lesion center display a more anti-inflammatory phenotype compared to foamy phagocytes in the lesion rim [18]. Vogel extended these findings by showing the majority of foam cells within MS lesions have an intermediate activation status, expressing markers that are characteristic for both inflammatory and anti-inflammatory phagocytes [200]. Collectively, these findings indicate that foamy macrophages showing an M2-like phenotype are apparent in both MS and atherosclerotic lesions, and that alike nuclear receptor signaling pathways drive the formation of these cells. Hence, identifying ways to specifically target these pathways in phagocytes will open therapeutic avenues for both MS and atherosclerosis.

## Conclusions and future perspectives

Our understanding of the role of foamy phagocytes in the pathophysiology of MS has increased tremendously over the last few years. It is becoming clear that the uptake of myelin by phagocytes is not merely a disease-promoting process but also a prerequisite for CNS repair. By simply inhibiting the uptake of myelin or reducing the number of lesional phagocytes, one will suppress the clearance of inhibitory myelin and counteract the protective phenotype that phagocytes adopt upon myelin internalization. To illustrate, the majority of therapeutics for MS are based on the assumption that prevention of immune cell infiltration into the CNS or elimination of them altogether is key to stop MS disease progression. While these therapeutics effectively reduce disease severity in early disease stages, they do not prevent lesion progression or promote CNS repair. Interventions that focus on enhancing the mobilization of phagocytes subtypes that are advantageous and/or depleting those that are detrimental may be more effective. For this purpose, naturally-occurring reparative processes should be exploited as our body has designed these processes to act accordingly. The uptake of myelin debris and the protective impact that myelin internalization has on the phenotype of phagocytes represent such processes.

Despite of the presence of protective foamy phagocytes, the majority of MS lesions evolve into chronic lesions as disease progresses. To date, it remains elusive what causes failure of foamy phagocytes to stop lesion progression and promote CNS repair as disease advances. Aging impacts immune cell function and recent evidence indicates that aging drives foamy phagocytes towards an inflammatory phenotype. Although MS is generally not regarded an age-related disorder, chronic inflammation might well lead to premature innate immunosenescence in MS patients. At the same time, valuable lessons can be learned from foamy macrophages in atherosclerosis and other diseases. As delineated in this review, ample evidence indicates that checkpoints involved in lipid handling are malfunctioning in macrophages following massive lipid uptake, leading to the intracellular accumulation of inflammatory lipids. Future studies should define whether faulty regulation of these pathways also occurs in mye-phagocytes. This could lead to the identification of new targets for therapeutic interventions and may open up new avenues for therapeutics currently used to treat other disorders characterized by the presence of foamy macrophages.
